# Dosimetric characterization and optimization of a customized Stanford Total Skin Electron Irradiation (TSEI) technique

**DOI:** 10.1120/jacmp.v14i5.4388

**Published:** 2013-09-06

**Authors:** Felipe Lučić, Beatriz Sánchez‐Nieto, Paola Caprile, Gabriel Zelada, Karen Goset

**Affiliations:** ^1^ Servicio de Radioterapia Clínica Alemana de Santiago Santiago Chile; ^2^ Instituto de Fisica Pontificia Universidad Católica de Chile Santiago Chile

**Keywords:** total skin electron irradiation, Stanford technique, mycosis fungoides, dosimetry

## Abstract

Total skin electron irradiation (TSEI) has been used as a treatment for mycosis fungoides. Our center has implemented a modified Stanford technique with six pairs of 6 MeV adjacent electron beams, incident perpendicularly on the patient who remains lying on a translational platform, at 200 cm from the source. The purpose of this study is to perform a dosimetric characterization of this technique and to investigate its optimization in terms of energy characteristics, extension, and uniformity of the treatment field. In order to improve the homogeneity of the distribution, a custom‐made polyester filter of variable thickness and a uniform PMMA degrader plate were used. It was found that the characteristics of a 9 MeV beam with an 8 mm thick degrader were similar to those of the 6 MeV beam without filter, but with an increased surface dose. The combination of the degrader and the polyester filter improved the uniformity of the distribution along the dual field (180 cm long), increasing the dose at the borders of field by 43%. The optimum angles for the pair of beams were ± 27°. This configuration avoided displacement of the patient, and reduced the treatment time and the positioning problems related to the abutting superior and inferior fields. Dose distributions in the transversal plane were measured for the six incidences of the Stanford technique with film dosimetry in an anthropomorphic pelvic phantom. This was performed for the optimized treatment and compared with the previously implemented technique. The comparison showed an increased superficial dose and improved uniformity of the 85% isodose curve coverage for the optimized technique.

PACS numbers: 87.53.Bn, 87.55.ne, 87.56.bd

## I. INTRODUCTION

The total skin electron irradiation (TSEI) technique started being used for the treatment of mycosis fungoides in the 1950s. The characteristics of the interaction between tissue and megavoltage electron beams make them favorable to be used for this purpose,^(^ ^1^ ^,^ ^2^ ^)^ as they are more penetrating than other therapies (e.g., phototherapy) and due to the fact that the dose falls rapidly after a few millimeters of tissue penetration for combined beams (oblique incidence).

**Figure 1:**
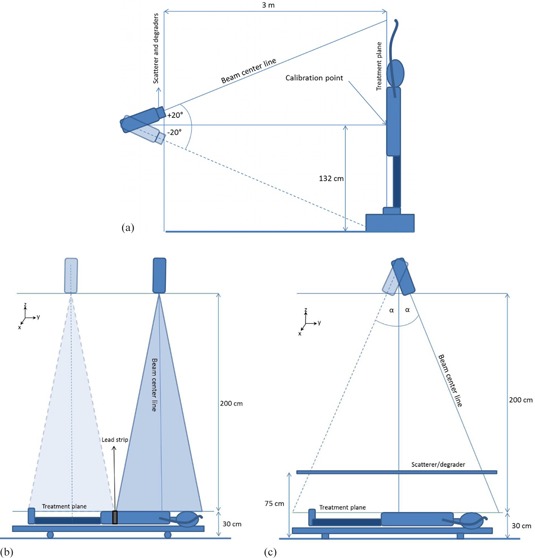
Patient position (relative to the linac head) for: (a) TSEI treatment using the Stanford technique, (b) the modified Stanford technique implemented in our institution, and (c) the configuration with angled beams.

High uniform dose to the skin and minimum dose to the internal organs is the goal of the TSEI treatment. This is not easy to achieve, due to the challenges imposed by the naturally irregular anatomy of the patient, which creates inhomogeneous dose distributions on the surface upon normal incidence of an electron beam. Since its first uses, different modalities of TSEI have been implemented clinically.^(^ ^3^ ^,^ ^4^ ^)^ The Stanford technique was developed by Karzmark and collaborators® and its main characteristics are: an extended source‐skin distance (> 300 cm), the use of double electron fields with symmetric angulations of ± 20° (as seen in Fig. 1(a)), and the application of the treatment at six standing patient positions (anterior, posterior, and four lateral oblique), corresponding to six angular dispositions of the patient (every 60°) with respect to the beam incidence. This technique has been considered as a safe and effective treatment, and has been selected by the majority of institutions in the last 20 years as the preferred modality.^(6)^ Several modifications from the original configuration have been implemented over the years, as attempts to improve the homogeneity of the distributions. These include the use of degraders,^(7)^ flattening filters,^(8)^ and lying down configurations, required when the patients are too frail to be treated in a standing modality.^(9)^

As can be noted, TSEI is a special technique that differs from the standard electron irradiation conditions where the beam configuration is close to the reference parameters established by recommended dosimetry protocols.^(10)^ The TSEI modality implemented at each center will depend on the equipment, facilities, training of the staff, and patient conditions.^(11)^ Taking into consideration the characteristics of this type of treatment, the importance of performing a complete dosimetric characterization under the specific treatment conditions is clear. The TSEI modality implemented in our institution was a modified Stanford technique, based on the use of six pairs of adjacent beams directed towards a translational platform, which supports the patient and facilitates the beam matching, as shown in Fig. 1(b).

The purpose of this study was to optimize the technique already implemented at our institution, in order to improve the homogeneity of the dose distributions obtained and to overcome the issues related to the matching of adjacent fields, shortening the treatment time and thus making it less uncomfortable for the patient. The procedure was carried out by investigating alternative electron energies, beam angles, and the use of custom‐made filters.

## II. MATERIALS AND METHODS

Electron beam irradiations were carried out using a GE Saturn 42 linac (General Electric Medical Systems, Buc, France). The dosimetric characterization of the beam was performed using a motorized water phantom WP700 (Scanditronix‐Wellhöfer, Uppsala, Sweden), a Farmer type ionization chamber (IC) model IC‐10 (Scanditronix‐Wellhöfer), and a NE‐2570/1 electrometer (Nuclear Enterprise, Reading, UK). In order to measure beam profiles and 2D dose distributions for the optimization of the technique, a set of 10 p‐Si diodes model EDD‐2 (Scanditronix‐Wellhöfer) and EDR‐2 films (Eastman Kodak Co., Rochester, NY) were used. The latter were developed using a Kodak X‐OMAT 1000 processor with an automatic chemicals mixer. A VIDAR scanner and software RIT 113 V4 (VIDAR Systems/Contex Group, Stockholm, Sweden) were used to digitalize and analyze the films. Absolute ionization chamber measurements were performed to calibrate the films.

Hereon, we describe the TSEI technique already implemented in our center, as well as the beam characterization procedures and optimization process.

### A. Modified Stanford technique

Our center has implemented a modified Stanford technique for the TSEI treatments. This modality relies on the use of a 6 MeV electron beam that is directed to the patient, who is lying down in a translational platform of $100\times 200\;{\text{cm}}^{2}$ at a source‐to‐surface distance (SSD) of approximately 2 m, as shown in Fig. 1(b). The collimators are set to define a $40\times 40\;{\text{cm}}^{2}$ field at the isocenter, producing a field size of $80\times 80\;{\text{cm}}^{2}$ at 200 cm from the source, sufficient to cover the transversal extension of an adult patient's thorax. This modality is preferred because it allows better positioning accuracy and reproducibility for frail patients (most treatments were palliative) undergoing a TSEI treatment.

The superior and inferior parts of the body are irradiated by adding two vertical beams, with parallel central axes separated by 80 cm, in order to obtain a treatment plane of $80\times 160\;{\text{cm}}^{2}$. This is done for each of the six patient positions (beam incidences) described in the Stanford technique.

To avoid hot spots at the region where the superior and inferior fields overlap, the line corresponding to the projection of the border of the light beam (simulating the irradiation field) is marked on the patient surface and a strip of lead of 3 mm thickness is used to match this line for each field. The line is shifted at each fraction to smooth the effect.

Treatment planning is performed manually. First, the average thickness of the anatomical sites of interest (head, thorax, abdomen, pelvis, thighs, knees, and ankles) corresponding to each field is determined for every patient position (relative to the beam incidence). Then, the number of monitor units (MUs) to be delivered is estimated by using the average thickness information for each field and assuming a homogeneous medium of water‐equivalent density. The dose is delivered at a constant rate of 400 MU/min.

### B. Dosimetric characterization

An electron beam, for a fix field size, can be characterized in terms of its percentage depth dose (PDD) curve and its related range and energy parameters. In order to define the appropriate patient positioning when using dual fields, it is important to know, in addition to the PDD, the characteristics of the longitudinal dose distribution of the composed field, which limits the extension of the usable beam.

#### B.1 PDD

The characterization of the single electron beam was performed, as mentioned before, by using the IC with the water phantom. This water tank was positioned at 200 cm SSD and filled with 30 cm of water, to simulate an average patient thickness. The dose and mean energy at the surface ($D_{s}$ and $E_{0}$, respectively), the photon contamination contribution $\left(D_{x}\right)$ and the therapeutic range $\left(R_{85}\right)$ were determined.

#### B.2 Profiles

It is important to know, in addition to the PDD, the extension of the usable beam (50% isodose). Transversal profiles (x‐axis, Fig. 1) were measured with the chamber at different depths in water. Due to the extension of the field at this extended SSD (80 × 80 cm light field at 2 m from the source), the water tank was shifted in the transversal direction twice to complete the profile.

To evaluate the longitudinal extension and uniformity of the dual field, a set of ten silicon diodes was used to measure relative profiles on the treatment plane. For this purpose, a rectangular acrylic phantom, positioned and irradiated according to the clinical specifications of the TSEI technique implemented (adjacent fields), was used. The phantom dimensions were $180\;\times \;120\;\times \;8\;{\text{cm}}^{3}$, providing sufficient backscatter material to ensure charge particle equilibrium at the measurement point. The diodes were located at the surface of the phantom in the transversal axis separated by 8 cm. These profiles were measured along the longitudinal axis (y‐axis, Fig. 1), at variable distances. Steps of 8 cm at the borders of the phantom and of 1.5 cm in the central 17 cm were used to cover 200 cm in total, defining a dose evaluation grid. This way, a 2D dose distribution grid at the treatment plane was established. Measurements in the beam junction were made under the lead protection sheet used for the treatments.

### C. Beam optimization

#### C.1 Energy

The first optimization criterion was related with the PDD and thus to the energy characteristics of the beam reaching the patient. The curves were measured with and without an acrylic degrader for nominal energies of 6, 9, 12, and 15 MeV. This degrader $\left(180\;\times \;120\;\times \;0.12\;{\text{cm}}^{3}\right)$ was introduced to improve the uniformity of the beam and to increase the superficial dose. It was positioned between the source and the patient (at 75 cm from the floor, equivalent to 155 cm from the virtual source). Two degrader thicknesses were evaluated, 8 and 12 mm.

The criterion to select the optimized beam was to preserve the therapeutic range of the 6 MeV clinical beam previously implemented, obtaining a superficial (or surface) dose as large as possible, without increasing the photon contamination contribution and the number of MU necessary to deliver the same dose.

#### C.2 Field length

Using the optimized nominal electron energy selected, a second optimization parameter was studied. This was motivated by the challenges related to the adjustment of the beam junction and consequent uncertainty in the delivered dose to this region when using the implemented technique; thus, a different approach was investigated. The optimized technique would no longer involve the translation of the patient; instead, the patient would remain lying at floor level and the gantry will rotate at symmetric angles to deliver two static superposed fields. The new configuration, shown in Fig. 1(c), allows the irradiation of the whole body of the patient. This reduces the treatment time by eliminating the repositioning procedure for the delivery of the second field, making the dose distributions at the center section of the body more reproducible and uniform than they were in the case of abutting fields.

The studied range of possible beam angles was from 18° to 27°. The optimum value was selected, trying to maximize the field length and considering the uniformity of the beam in the central section of the patient.

Measurements were carried out with the set of diodes located in the longitudinal axis of the dose evaluation grid, as described above in B.2.

#### C.3 Field uniformity

**Figure 2:**
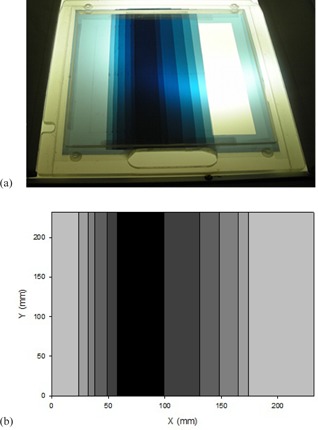
Custom‐made polyester flattening filter (a) used to homogenize the longitudinal beam profile; (b) schematic scaled representation of the filter. Note that the maximum thickness does not correspond to the center of the field, as it is meant to be used in an angled beam configuration.

In order to further improve the homogeneity of the fields at the patient surface, for the angled configuration, a special custom made filter was designed. This flattening filter, shown in Fig. 2, was made of several layers of polyester. The sheets of polyester were obtained by developing blank films, and were mounted in an acrylic frame. The material was selected because of its availability and its low Z value, which reduced the probability of generating bremsstrahlung. The filter was designed to be thickest at a shifted position from the geometrical center of the field, taking into consideration that the treatment was carried out by a superposition of two angled beams.

Ten diodes were used to measure the relative profile at the treatment plane of a single angled beam. The profile was back‐projected to the tray plane, providing a first guess for the filter design. Then, the process was repeated, until an optimum configuration was reached. The border of the field was only slightly filtered, in order to preserve the particle fluence at the superior and inferior edges of the beam (the first sheet of polyester covered the entire exit window as a base for the additional sheets). As only one filter was build and mounted, the linac head needed to be rotated 180° for the second (symmetric) beam angle. The final configuration of the filter (sheets dimension and position) is shown in Table 1.

**Table 1:** Characteristics of the flattening filter made of polyester

*Sheet No.*	$\text{Dimensions XxY}\;({\text{cm}}^{2})$	*X Distance from the Field Edge (cm)*	*Accumulated Polyester Thickness (mm)*
1	23.2×23.2	0	0.15
2	23.2×15	2.4	0.30
3	23.2×13	3.2	0.45
4	23.2×11	3.8	0.60
5	23.2×8.2	4.9	0.75
6	23.2×5	5.8	0.90
7	23.2×3.5	6.4	1.05

### D. Optimized Technique

After the optimal combination for the dual electron field was found, 2D transversal dose distributions measurements were performed. This was done using film dosimetry and an anthropomorphic (pelvic) phantom irradiated under clinical TSEI conditions. In order to evaluate the contribution from the different beams to the final AP depth dose of the complete treatment, the phantom was irradiated using a single optimized dual beam incident from the AP direction, then from the two anterior‐oblique directions, and finally from the remaining PA position, to complete the six positions described by the Stanford technique. Additionally, the previously implemented modified Stanford technique was also used to irradiate the phantom, allowing the comparison of both treatments.

## III. RESULTS

Hereon we present the results of the optimization of the TSEI treatment, comparing the characteristics of the optimized technique with the implemented modified Stanford technique. This is done for the single incidence dual field, as well as for the complete treatment.

### A. Dual beam characteristics

Table 2 shows the result of the beam characterization under TSEI conditions (extended SSD of 200 cm) for different energies and degraders. It can be noted that, as the degrader thickness increases, the superficial dose grows, along with a decrease in $R_{85}$. A slight but consistent increase in the Bremsstrahlung contamination with respect to the configuration without filter can be noted for the 8 mm filter; however, for the 12 mm filter the effect is not observed systematically.

**Table 2:** Beam parameters measured at 200 cm SSD for an $80\times 80\;{\text{cm}}^{2}$ field size at the surface

	*Nominal Energy (MeV)*	*6*	*9*	*12*	*15*
No acrylic flter	$E_{0}\;(\text{MeV})$	5.6	8.23	10.57	13.44
	$D_{S}\;(\%)$	80.9	82.2	83.8	86.7
	$D_{X}\;(\%)$	0.8	1.2	1.4	1.7
	$R_{85}\;(\text{mm})$	18.9	28.5	36.9	47.8
8 mm acrylic flter	$E_{0}\;(\text{MeV})$	3.69	6.03	8.73	12.24
	$D_{S}\;(\%)$	91.3	88.4	89.1	90.5
	$D_{X}\;(\%)$	1.4	1.5	1.8	2.4
	$R_{85}\;(\text{mm})$	11.1	19.7	29.4	38.7
12 mm acrylic flter	$E_{0}\;(\text{MeV})$	2.98	5.58	7.87	10.65
	$D_{S}\;(\%)$	96.5	90.9	90.9	91.4
	$D_{X}\;(\%)$	1.4	1.2	1.3	1.6
	$R_{85}\;(\text{mm})$	8.4	17.5	25.9	36.1

The isodose curves in the transversal plane for a 9 MeV beam with an 8 mm degrader were used to evaluate the extension of the usable field (50% isodose curve). It was found that at $R_{85}$ the width of the dual field was of 74 cm.

**Figure 3:**
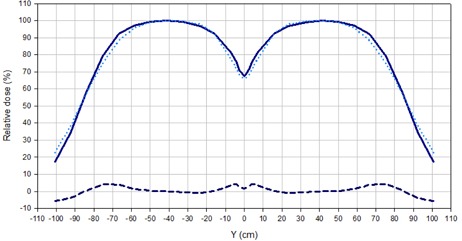
Comparison of longitudinal profiles for the adjacent field technique, normalized to 100% at the center of each field. The solid line corresponds to a 6 MeV beam without filter and the dotted line to a 9 MeV beam with 8 mm acrylic degrader. The dashed line indicates the difference.

Diode measurements along the longitudinal profile for the adjacent field technique showed that the dose under the lead protection was reduced for each field to a 3.4% of the central axis dose in the case of a 6 MeV beam without filter, and to a 4.3% in the case of a 9 MeV beam filtered by the 8 mm degrader. The complete longitudinal profile was determined assuming symmetry of the distributions for the superior‐inferior fields. Figure 3 shows the comparison between longitudinal profiles of the two beam settings. As can be seen, the differences remained under ± 5% for the central 180 cm of the composed field. Underdosages at the center of the dual field, with respect to the maximum dose, of 32% for the 6 MeV and of 34% for the 9 MeV (with filter) beam were found.

Considering the results shown in Table 2 and Fig. 3 and the MUs required to obtain the same detector signal at the maximum, a beam setting of 9 MeV with 8 mm degrader was chosen as the optimized energy/filter setting.

A continuous loss of uniformity in the central section of the composed field was observed for angles greater than 24°. However, for 24° the homogeneous region was very limited (100 cm long within ± 5% and 120 cm within ± 10%), dropping to only around 60% of the dose in the center at ± 90 cm from the central axis. For 27°, the central part of the field was not uniform; nevertheless, the dose at ± 90 cm from the center was close to 90% of the dose at the central axis.

**Figure 4:**
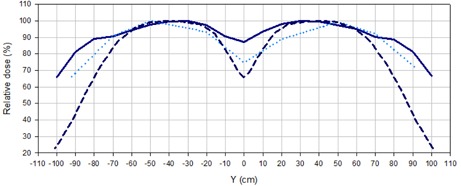
Longitudinal profiles for 9 MeV beams with the 8 mm acrylic filter measured with diodes for different delivery techniques: adjacent beams (dashed line), 27° angled beams (dotted lines), and 27° angled beams with added flattening filter (solid line).

The 27° angle was chosen and the additional custom‐made flattening filter, described in C.3, was added to improve the uniformity in the central part of the field. The design of the added flattening filter permitted a reduction in the dose gradient with respect to the adjacent beam configuration, while increasing the dose at the borders of the central 180 cm length region by 43% with respect to the previous implementation. The comparison shown in Fig. 4, illustrates this effect.

### B. Composite Distributions for the Optimized Technique

**Figure 5:**
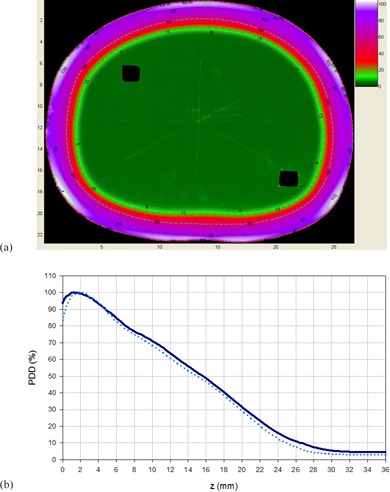
Transversal relative dose distribution (a) obtained from a film irradiated with 6 dual fields of 9 MeV beam with degrader and added flattening filter, normalized to the dose at 12.2 mm depth along the AP axis (corresponding to $R_{100}$ of the AP field); (b) composite percentage depth dose distribution for the 6 dual fields with the modified Stanford technique (dashed line) and the optimized technique (solid line).

The relative dose distribution obtained as a result of the superposition of the optimized beams from the six incident directions in an anthropomorphic phantom is shown in Fig. 5(a). It shows how the 85% isodose curve completely encompasses the surface of the phantom. A PDD was extracted from the film measurement and compared with the PDD obtained for the already implemented technique. Figure 5(b) shows the comparison, where the increase in the dose at the surface and photon contamination for the new technique can be noted.

**Figure 6:**
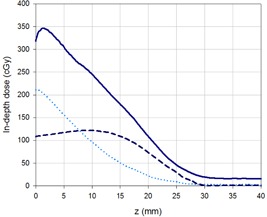
In‐depth dose distributions measured at the AP axis for the contributions of: AP dual field (dashed line), anterior‐oblique fields (dotted line), and the complete treatment (solid line). The planned dose was of 100 cGy at $R_{85}$ for each dual field.

Figure 6 shows depth dose curves measured at the AP axis with film dosimetry in the anthropomorphic phantom, considering the contributions of different composed fields of the optimized TSEI treatment (AP and anterior‐oblique). It can be noted that the contribution to the surface dose (and down to 2 mm depth) of the superposition of AP and anterior‐oblique fields triples the contribution from the AP field alone, as the contribution from the anterior‐oblique fields doubles the one from the AP. When the distributions are normalized to the maximum, the PDD of the complete optimized treatment showed the expected shifting of the maximum dose toward the surface and an increase in $D_{s}$ of 10%, due to the superposition of the fields, increasing the photon contamination to 4.2%.

### C. Clinical implementation

**Figure 7:**
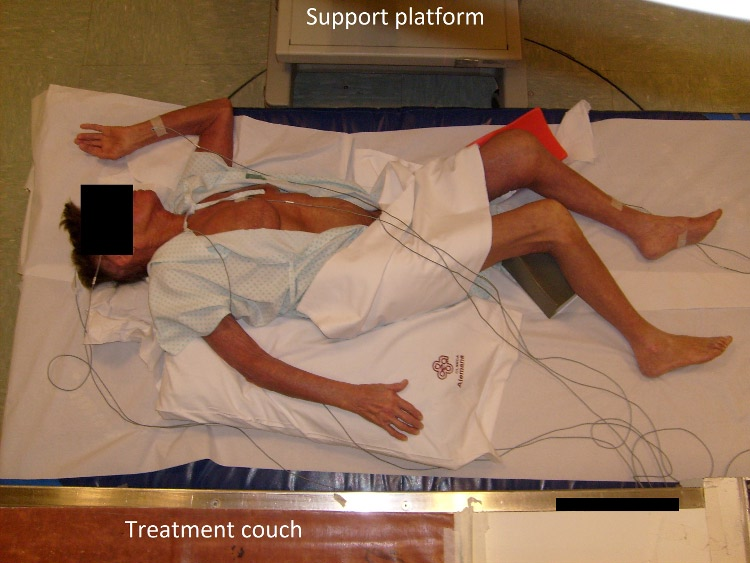
Patient positioned for an anterior‐oblique irradiation. Pillows and foam wedges help patient hold the position. An *in vivo* dosimetry system is used to monitor treatment delivery. The acrylic degrader (not seen in this picture) is placed above the patient, using the support platform and the treatment couch seen at the top and bottom of the picture, respectively.

Our modified Stanford technique uses a lying down positioning; this has clear advantages for weak patients. However, the application of oblique fields has challenges related to reproducibility and patient comfort. In order to address this issue, pillows and foam wedges are used. Figure 7 shows the patient positioned for an anterior‐oblique incidence.

As can be noted, *in vivo* dosimetry based on semiconductor diodes is used to monitor dose delivery, including the X‐ray contamination contribution. Monitored points corresponded to the center of the field and regions of reduced diameter and/or at the borders of the treatment field (ankles, wrists, scalp, and sole of the foot). The decision of additional boosts or protection of any of these anatomical regions was made based on such measurements.

## IV. DISCUSSION & CONCLUSIONS

One of the objectives of this study was to find a new setting of the beam, in which the superficial dose is increased, but the therapeutic range $\left(R_{85}\right)$ remains as close as possible to the one from the modified Stanford technique already in use (6 MeV nominal energy without filter), limiting as much as possible the photon contamination. Considering the parameters shown in Table 2, it was found that the best compromise to fulfill these requirements was to use a 9 MeV beam either with the 8 or 12 mm degrader. However, the number of monitor units required to deliver the same dose using the 9 MeV beam with the 12 mm degrader, compared with the ones needed for the 8 mm filter, were significantly higher. Using this combination would then increase the treatment time, which would affect patient comfort, introducing more uncertainties related to patient positioning. As both techniques were able to fulfill the requirements imposed in terms of beam characteristics, but the thinner filter implied fewer MUs, the 9 MeV beam with the 8 mm filter was selected to be used in the optimized technique. This setting allowed increasing the superficial dose from 80.9% to 88.4% of the maximum, with an increase of 0.8 mm in $R_{85}$, while keeping photon contamination at a low level, increasing only in a 0.9% of the maximum dose. The isodose distribution at $R_{85}$ for the selected setting was considered to be appropriate for the TSEI treatment, as the width of the 50% isodose curve of 74 cm would be sufficient to cover the transversal extension of an adult patient's thorax.

Although the selection of an optimized beam/filter setting produces the desired effect of increasing the dose to the surface, it does not solve the previously mentioned problems. Thus, the use of angled beams provides the ideal approach to reduce the uncertainties in the total treatment dose received by the patient at the central region. Additionally, it extends the range of the useful beam in the longitudinal direction and reduces the treatment time (by eliminating patient repositioning).

Variations in the uniformity of the beam across the longitudinal profile for the selected beam setting and different symmetric gantry angulations resulted in two expected effects. As the angle increased, a loss of uniformity in the central part of the dual field, accompanied by an increase of the field extension, was observed. Within the studied angle range, it was found that the best uniformity at the central region was achieved by the 24° angle. Under this configuration, the dose close to the edges of the field was reduced to 60% of the central dose. As this is considered insufficient to treat the full extension of an adult patient, a greater symmetric angle was needed to optimize the beam. Symmetric angles of 27° provided a useful field of 180 cm in the longitudinal direction, considered sufficient to treat the great majority of the patients. However, the uniformity of the dose across the field was greatly compromised by the increase in the obliquity of the beam incidence. Due to the fact that the uniformity of the beam can be further improved by the addition of a flattening filter, the 27° angle was selected regardless, prioritizing the extension of the usable beam.

The clinical beam optimized by the use of an energy of 9 MeV with a degrader, an added filter, and a symmetric rotation of the gantry in ± 27° for the subfields composing beam, allows the treatment of taller patients than it was possible with the adjacent beam technique. Furthermore, beam uniformity was improved, giving more flexibility to position the patient and reducing the treatment time. The gradient of the longitudinal profile obtained was close to the results reported by Chen et al..^(11)^

In Figure 5, the final transversal dose distribution obtained for the optimized TSEI treatment delivered to a phantom can be seen. Although the surface dose presents inhomogeneities (the directions of incidence are clearly noted), the 85% isodose encloses the patient's surface in a uniform way. The increase in photon contamination (to a total of 4.2%), due to the superposition of fields for the complete TSEI treatment, remains within acceptable limits recommended by the AAPM for composite electron beams.^(3)^

The AAPM^(3)^ defines a B factor (overlapping factor) that can be obtained as the ratio between the mean surface dose in a cylindrical (30 cm diameter) phantom for the complete treatment, and the surface dose for a single dual field. Figure 6 shows the PDDs that allow the estimation of this factor; in this case, each composed field contributes with one third of the superficial dose. The use of factor B allows simplifying the manual treatment planning, as the dosimetry of only one field would be sufficient to estimate the dose deposition at the surface of the patient due to the contributions of the six dual fields. The factor obtained for our optimized implementation was 3, consistent with the range reported by the AAPM (from 2.5 to 3) for Stanford type TSEI techniques.^(3)^ Due to the increase in the obliquity of the beam incidence over structures with reduced diameter (as arms and legs), an increase of the overlapping factor under these conditions would be expected for any implementation of a Stanford technique. This effect could explain short‐ (epithelitis) and long‐term (formation of fibrotic zones and telangiectasias) unwanted reactions observed in the extremities of some patients treated at our center, and should be considered when planning the treatment.
